# The Mental Health Implications of Domestic Violence During COVID-19

**DOI:** 10.3389/ijph.2021.1604240

**Published:** 2022-01-21

**Authors:** Elizabeth A. Newnham, Yanyu Chen, Lisa Gibbs, Peta L. Dzidic, Bhushan Guragain, Satchit Balsari, Enrique L. P. Mergelsberg, Jennifer Leaning

**Affiliations:** ^1^ School of Population Health, Curtin University, Perth, WA, Australia; ^2^ FXB Center for Health and Human Rights, Harvard University, Boston, MA, United States; ^3^ Melbourne School of Population and Global Health, University of Melbourne, Melbourne, VIC, Australia; ^4^ Centre for Victims of Torture, Kathmandu, Nepal

**Keywords:** mental health, COVID-19, domestic violence, complex PTSD, mental health services

The COVID-19 pandemic has magnified conditions for trauma, stress, financial insecurity, and isolation; each known to have unique and cumulative effects in exacerbating the frequency and severity of domestic violence [1]. Large-scale public health measures, such as physical distancing and lockdowns, have reduced COVID-19 transmission but paradoxically created conditions for domestic violence perpetrators to exercise increased financial, physical and psychological control [1, 2]. Isolation at home means that many survivors of domestic violence are unable to access telephone helplines, services, finance, informal social supports or safe shelter [2, 3]. Similarly, the closure of schools has resulted in children spending significantly more time at home than usual, placing them at greater risk of witnessing and/or experiencing any violence occurring in their homes. Domestic violence has enduring effects for mental health [4], and will create a significant increased need for trauma-informed services both during and after the COVID-19 pandemic.

## Rising Prevalence of Domestic Violence

There is global evidence of rising rates and intensity of domestic violence during periods of physical distancing and lockdown [2, 3, 5–7]. Elevated reports of incidence and help-seeking have been consistent across nations, and the data are likely to be conservative given the increased difficulty for survivors to report abuse during pandemic response periods [2]. Significant gaps in data collection and reporting have limited capacity to synthesize findings and determine comparative levels of risk, thus hindering a coordinated global response. However, the weight of evidence indicates that we are facing a global crisis in domestic violence that places substantial numbers of women, children and men at risk of harm and death.

## Mental Health Consequences of Domestic Violence

The mental health effects of domestic violence are likely to be severe and long-lasting [4]. Exposure to violence and abuse increase one’s risk of experiencing post-traumatic stress disorder, depression, anxiety, substance use, and suicidal behaviours [4, 8]. The most commonly employed element of domestic violence–coercive control—is a pattern of domination enacted through tactics designed for intimidation and entrapment, and has particularly damaging effects for mental health [2, 6]. Coercive control strategies include social and physical isolation, shaming and belittlement, micromanagement of daily activities, and constant surveillance. These strategies aim to terrorise, hurt and overwhelm victims, and predict intimate partner homicide [9]. Control strategies may differ by culture and setting, and during the COVID-19 pandemic perpetrators are capitalising on the isolating conditions of lockdowns and home quarantine to enforce separations from social support networks, increase control of victims’ actions and finances, and exacerbate fear within the household [1, 3, 10]. Practitioners are also seeing the use of digital coercive control, whereby perpetrators utilise technology to monitor and track their victims, creating a sense of omnipresence, isolation, and ostracism [9]. Coercive control has significant implications for survivors’ mental health, through prolonged, repeated trauma that is both inescapable and unpredictable in nature.

Chronic and repeated trauma often manifests a more complex pattern of psychological symptoms compared with a single trauma event [11]. In addition to the defined symptoms of post-traumatic stress disorder (PTSD), people with complex PTSD are more likely to experience dissociation; alterations in memory, identity, and personality; negative self-concept; disturbances in relationships and impaired functioning [11]. Similarities in the pattern of psychological symptoms resulting from domestic violence are evident across cultures, with large-scale community surveys indicating that women who have experienced domestic violence are at higher risk of complex mental health difficulties and suicidal thoughts than women who had not experienced violence [2, 12]. Mental health difficulties disrupt economic engagement and livelihoods, which will have significant implications for post-COVID economic recovery. A growing mental health crisis—triggered by pandemic stressors, infection and compounded by domestic violence—will thus require specialised trauma-informed services.

## The Role of Mental Health Service Providers

Addressing domestic violence requires a multi-pronged approach involving trauma-informed and culturally secure legal services and policing, tailored mental health services, and broad societal efforts [8]. As we transition into new stages of the pandemic, mental health services must be prepared for an influx in demand and caseload, with required attention to the psychological impacts of trauma. In high-income settings, it is vital that psychologists, psychiatrists, mental health nurses and general practitioners are provided sufficient training to identify the incidence and mental health effects of domestic violence, and that clinicians are confident and capable in delivering evidence-based, trauma-informed care that is culturally secure and tailored to the unique circumstances of the pandemic. The implementation of trauma-informed practices in domestic violence services has demonstrated significant improvements in women’s safety-related empowerment and self-efficacy [13].

In low-income settings, where mental health resources are scarce, task shifting will be an important priority for government and non-government services. Task shifting involves the training and ongoing supervision of lay-providers to enable competent delivery of psychological interventions in areas with few mental health professionals. Culturally-adapted psychological interventions are effective in improving psychological symptoms when delivered by trained lay-providers, fostering a broader coverage of mental healthcare while improving cost-efficiencies, reducing mental health stigma and creating employment opportunities in low-resource settings [14]. Training lay-providers to deliver mental health programs for people affected by domestic violence and abuse during the pandemic will support broader treatment accessibility and improve prevention efforts, particularly in rural and remote communities where isolation intensifies the risk of trauma [14]. Remote and asynchronous learning can also help accelerate the training of lay-providers to meet the anticipated large demand [15]. Further, the implementation of telehealth services will expand treatment coverage, enabling greater access to mental health providers [3], while reducing transmission risk for providers and the community. During lockdowns, the continual presence of the perpetrator within the home will restrict survivors’ ability to discuss their situation via phone; however telehealth and online services have potential to reduce isolation, disseminate safety information, and enable safety planning and referrals via coded messages and disguised phone apps [3, 16].

### Conclusion

The compounding mental health impacts of stress, infection and violence have created acute strains on mental health systems worldwide, requiring significant investment and innovation by services and governments. Mental health services must immediately coordinate efforts to scale up training of professionals and lay-providers and establish sustainable systems for culturally-secure, trauma-informed mental healthcare. Ongoing economic investment in established domestic violence services and growth of the mental health sector will be critical in supporting violence survivors throughout the pandemic.
